# Thermodynamic Rarity and Recyclability of Raw Materials in the Energy Transition: The Need for an In-Spiral Economy

**DOI:** 10.3390/e21090873

**Published:** 2019-09-08

**Authors:** Antonio Valero, Alicia Valero

**Affiliations:** Instituto CIRCE (Research Centre for Energy Resources and Consumption), Universidad de Zaragoza, 50018 Zaragoza, Spain

**Keywords:** Thanatia, rarity, recyclability, critical raw materials, energy transition, circular economy

## Abstract

This paper presents a thermodynamic vision of the depletion of mineral resources. It demonstrates how raw materials can be better assessed using exergy, based on thermodynamic rarity, which considers scarcity in the crust and energy requirements for extracting and refining minerals. An exergy analysis of the energy transition reveals that, to approach a decarbonized economy by 2050, mineral exergy must be greater than that of fossil fuels, nuclear energy, and even all renewables. This is because clean technologies require huge amounts of many different raw materials. The rapid exhaustion of mines necessitates an increase in recycling and reuse, that is, a “circular economy”. As seen in the automobile industry, society is far removed from closing even the first cycle, and absolute circularity does not exist. The Second Law dictates that, in each cycle, some quantity and quality of materials is unavoidably lost (there are no circles, but spirals). For a rigorous recyclability analysis, we elaborate the exergy indicators to be used in the assessment of the true circularity of recycling processes. We aim to strive toward an advanced economy focused on separating techniques and promoting circularity audits, an economy that inspires new solutions: an in-spiral economy.

## 1. A Second Law Vision of Planet Earth

The Second Law deals with evolution, irreversibility, and degradation, key tenets for understanding sustainability. In the current study, we philosophically and conceptually examine how this law can assess the depletion of natural resources, especially minerals, based on indicators and published examples. We also develop new exergy-based indicators to assess the circularity of recycling processes. Thus, it is important to first present a “Second Law vision” of the Earth through a global exergy analysis. Our study expands upon an earlier extended keynote lecture at ECOS 2018, Guimaraes, Portugal.

Exergy is a property of a system relative to an associated reference state. It is the maximum work a system can deliver as it interacts with another large, but real, system, namely, a reservoir. Such a reservoir attracts the system toward degradation or entropy creation.

The Earth is a sunbathed celestial ball floating in the Universe. Over billions of years, the Sun has provided the energy required to shape the Earth into its current conditions. The Sun’s energy is radiated daily into the Universe, where the temperature is around 2.7 K (−270.4 °C). Only 0.024% of the Sun’s radiation is stored through photosynthesis—this fraction, in addition to the weathering effects of the Sun’s energy on the crust as well as the internal heat on Earth, shaped the conditions for the creation and storage of all biological and mineral resources humanity has at its disposal for survival.

A physical observation of the Sun–Earth system allows us to observe spontaneous processes such as erosion, oxidation, mixing, cooling, melting, evaporation and precipitation, mass transport, and dispersion of materials. There exist anthropogenic processes such as extraction and use of natural resources; solid, liquid, and gas pollution; and climate change. Given adequate time, the Sun, along with the internal heat of Earth’s inner cores, could provide the energy to partly restore irreversible degradation. However, the pace of such degradation is different for biological systems than for geological ones. Biological systems may be restored in decades or centuries, whereas geological resources are non-renewable within the human timescale. Contrary to anthropogenic processes, which are usually linear, natural processes behave cyclically. Nature evolves at its own complex pace and cannot restore what humans hastily dispose. The anthropogenic effects on this evolution are so high in magnitude that our era is now called the Anthropocene era [1].

We now consider the exponential anthropogenic depletion of non-renewable natural resources either from an economic or from a thermodynamic point-of-view.

From an economic point-of-view, it is easy to extrapolate and hypothesize a planet without (some or all) economically available mineral deposits, irrespective of the time it would take to reach such extreme conditions. Such a moment would be a tipping point, where the global decline of the marginal benefit of mining would meet the increasing marginal cost of energy and its environmental impact [2]. This economic view is a realistic possibility in the near future, but it has no means to assess which resource would deplete first. It offers certitude in the collapse, but incertitude in identifying the causative factor.

From a thermodynamic point-of-view, suppose an initial state, which is the beginning of the Anthropocene. At this point, the Earth is mature and natural resources are abundant. The anthropogenic depletion and dispersion of minerals as well as ecosystem destruction do not allow the Sun to restore these resources within the human timescale. This would drive the Earth to a greater entropic state. Let us name that final state “Thanatia” (from Greek *Thanatos*, meaning “death”) [3,4], that is, a planet without concentrated mineral deposits and fertile soils, with complete dilution of fresh waters into the sea, and the highest CO_2_ concentration in the atmosphere, comparable to the complete burning of all remaining fossil fuels [5,6]. This imaginary state of the planet does not need to be “reachable,” but it is a baseline to physically assess how close to depletion each natural resource is. It allows us to objectively identify which resource will be the first to deplete in the race to exhaustion.

Thanatia is an instrumental “dead state” of the Earth, and it is of great concern to humans. It is not a reference coordinate scheme to calculate chemical exergies, such as the established “reference environment” by Szargut [7]. In fact, it is a realistic possibility of entropic degradation. Essentially, Thanatia could signal the end of the Anthropocene era. It is meaningful to understand the current civilization using a countdown to an end, especially in the context of the aging of humanity. This best exemplifies the Second Law. Such an era would not be an absolute end of Earth but an epoch in its evolution.

Once this dead state is modeled, thermodynamics allows to assess the rate of exergy loss of our valuable natural resources. Any natural resource distinct from Thanatia would have exergy that could be measured in kilowatt hour (kW·h). Further, any material extraction from nature can be physically compared with its alternative recycling process to draw global, social, environmental, economic, and political implications. This paper presents a clear case of such conclusions and particularly demonstrates how the theory presented can be used to evaluate mineral resource use and depletion. We next present the authors’ approach derived from the theory of Thanatia [3]. It is important to state that other authors such as Stanek (thermoecological cost concept [8]), Sciubba (extended exergy analysis [9]), and Dewulf et al. [10,11] (Cumulative Exergy Extraction from the Natural Environment) have successfully developed and applied exergy indicators to resource depletion assessment.

## 2. Embodied Exergy, Exergy Replacement Cost, and Thermodynamic Rarity: Definitions and Precisions

A mineral deposit is a high-valued exergy resource that is physically separated from its surrounding environment because of its specific chemical composition, concentration, and cohesion degree with respect to the average upper crust, that is, Thanatia. Because minerals are usually concentrated in mines than dispersed across the crust, their extraction and beneficiation energy is lower. Nevertheless, the exergy of minerals is not a good indicator of its commercial value. We know that mixing processes seldom release exergy, and yet the reverse processes of separation are major exergy consumers [12]. This is also the case of crushing and grinding processes, where mining operations expend enormous quantities of exergy compared with the exergy release in spontaneous crystallization. Compared with fossil fuels, the chemical exergy of metallic oxides is almost negligible. Therefore, a better indicator of the physical value of a mineral cannot be only linked to its chemical exergy—rather, it is the concentration or scarcity of the mineral that makes it physically valuable. To consider the “irreversibilities” that occur in separation processes, the concentration exergy costs, rather than exergies only, need to be considered.

Accordingly, one could assess the physical value of a given mineral as the exergy cost required to mine, beneficiate, and refine minerals from mineral deposits (i.e., through so-called embodied exergies). However, in doing so, we would grant a lower value to high-grade mines, which are easier to mine. With extraction, high-grade mines become depleted, and the extraction exergy costs increase. Indeed, because minerals are concentrated in mineral deposits, we can avoid serious energy expenditures. We thus propose to assess the mineral capital in nature by considering not only embodied exergies, but also the avoided costs arising from having minerals concentrated in mines and not dispersed across the crust. Accordingly, thermodynamic rarity is defined as the amount of exergy resources needed to obtain a commodity from ordinary rocks (Thanatia) using the prevailing technology [13].

Thermodynamic rarity is the sum of two terms: An imaginary one that results from evaluating the avoided cost of obtaining the metal from ordinary rocks to its concentration and composition in the mine; and a second real term obtained as the embodied exergy or exergy cost from the mine to the commodity metal or mineral, that is, the exergy needed to extract, beneficiate, transport, smelt, refine, and manufacture the material/product to market. The imaginary part is called the exergy replacement cost. It is not spent in reality and can be obtained by simulating the mining operations required for hauling, crushing, and grinding ordinary rocks and then separating the required mineral from its gangue. In their studies, Valero and colleagues presented exergy replacement cost values [14,15].

The thermodynamic rarity indicator has interesting features. It implicitly considers the geological scarcity of any metal or element in the crust because, the lower the amount of the element content in Thanatia, the higher its rarity. It is a quantitative property of materials that can be measured in kW·h, similar to exergy. It is additive, and hence the rarity of a mixed material is the weighted average of the rarities of its components in addition to the exergy needed to produce it.

The rarity value of a product also depends on a fixed initial state—in this case, Thanatia, the final state, which refers to the market specifications of the product and its manufacturing technology. Often, the exergy costs of mining, beneficiation, and smelting are the dominant values of the manufacturing exergy expenses. Such primary operations have a slow pace in the optimization of energy consumption. Thus, rarity stabilizes for most metals and industrial minerals. As mines become depleted and ore grades approach crustal concentrations, it is the latent value of the imaginary part that slowly emerges.

The thermodynamic rarity indicator allows us to convert the debt left for future generations into exergy values. It includes two messages: conservation (through the exergy replacement costs) and process efficiencies (through real embodied exergies). Whereas the latter are widely recognized, the former is neglected primarily because of a lack of knowledge and indicators. Yet, both indicators are equally necessary for the rational management of resources because conservation entails avoided replacement. Indeed, one can associate a cost of replacement to each act of conservation, whether relating to mineral resources or any natural resource in general. The cost of replacement acts as a psychological barrier that prevents further deliberate destruction. Enhancing reuse and recycling can thus reduce further extraction (see Section 5). The more irreplaceable an object is, the stronger the desire to conserve it. In short, the exergy replacement cost represents the mineral capital stored in the crust that can be eventually used to move the economy, such as the coming “energy transition” (explained hereafter).

## 3. An Exergy Flow Analysis of the Energy Transition

**Figure 1:**
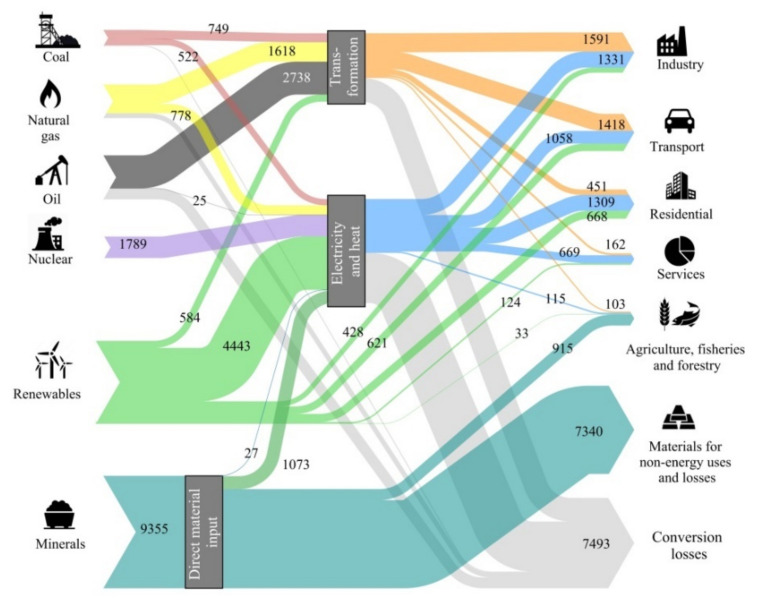
World exergy flow analysis for the International Energy Agency 2DS scenario for year 2050. All data are expressed in mega tonnes of oil equivalent (Mtoe) [19].

Energy sources can be very diluted, such as with solar or geothermal, or be highly concentrated, such as with fossil fuels or nuclear energy. Highly concentrated energy sources have complex problems. For example, fossil fuels have adverse effects on climate change, whereas nuclear energy processing requires treatment of dangerous waste and entails the risk of accidents. To counter these issues, a global agreement exists that nations must promote renewable energies. However, the efficient and intelligent use of energy requires equipment, which, in turn, requires raw materials. Today, some of these raw materials are already scarce in nature, posing important supply risk problems (i.e., critical). Renewable energy technologies and electric mobility are now more mineral intensive than current energy sources. Thus, to what extent can the world become dependent on these materials? Do we need sufficient critical raw materials to support an energy transition to combat climate change? For example, new efficient solar photovoltaic cells may contain copper, silver, silicon, cadmium, tellurium, indium, selenium, or gallium. Most wind turbines need permanent magnets with high contents of neodymium, praseodymium, and dysprosium with boron and iron or, alternatively, cobalt and samarium. They also require copper and other structural materials such as steel and aluminum. Biomass, on the other hand, requires phosphates, potassium, and other microelements such as zinc, magnesium, cobalt, and manganese. In Valero et al. [16], we published a list of elements that might be at risk because of supply shortages for the development of green technologies. We also estimated the mineral capital assessed in exergy terms that would be necessary to maintain the global temperate below 2 °C, as proposed in the Paris Agreement of the 21st Conference of the Parties [17]. Among the many long-term international projections, we took the 2DS scenario from the International Energy Agency [18] that accepts a global temperature increase of 2 °C with a probability of 50%, reaching the GHG peak in 2020 and decreasing gradually until reaching the neutrality of the energy sector in 2100. This scenario contemplates the growth of the population and the adoption of economic, energy, and environmental policies leading to an increased share of efficiency, renewable energies, and electric mobility according to the prefixed objectives. The result for such an expected energy transition is illustrated in Figure 1 as a Sankey diagram. To retain the same units, we evaluated each metal demand in terms of its exergy replacement cost rather than in tonnage. However, tonnage was also assessed as explained in Valero et al. [19].

The aforementioned study found that the energy transition as proposed under the 2DS scenario would entail a 30% increase in mineral demand, from 7193 Mtoe in 2025 to 9355 Mtoe in 2050. Five elements experience at least a six-fold increase in demand in exergy replacement cost terms—cobalt, lithium, magnesium, titanium, and zinc. Further, the growth in phosphates and potassium demand both for fertilizers and bioenergy use is expected to double by 2050 [19]. Figure 1 shows that to approach a decarbonized economy by 2050, the replacement cost of the minerals used (9355 Mtoe) is greater than the energy provided by all fossil fuels and nuclear energy (6405 + 1789 Mtoe) and even by all renewable energies (6901 Mtoe).

Just as in history, ore grades will decline [20,21,22]—in some minerals more than in others—and will cause replacement costs to become real energy costs. The minerals that are extracted with ease will become expensive to extract eventually. In fact, the exergy replacement cost helps us anticipate future costs. As the literature shows, the loss of mineral capital in future will be exorbitant [23,24,25].

On the other hand, the mining sector will in no way be marginal to the economy and the prices of raw materials will arguably increase sharply. In future, it will be challenging to carry out largescale land movements for mining with electricity produced by renewable energies rather than keeping common diesel trucks. Similar possibilities exist for smelting techniques, which require high temperatures that are difficult to attain with renewable sources. Therefore, it is likely that the mining and smelting sectors will increase their dependence on fossil fuels, at least in the short to medium term. In addition, the damage of extracting large quantities of minerals will seriously affect ecosystems as well as local populations that will increasingly become less willing to accept such adverse destruction.

Hence, the energy transition can only evolve with careful management of raw materials through a “circular economy.”

## 4. Toward a Circular Economy: The Case of the Automobile Sector

The most important concept in the Second Law is irreversibility, that is, the one-directional nature of real processes. The use of energy is inexorably linked with its degradation. Therefore, the problems energy engineers face focus on improving the efficiency of energy use. Unlike energy, materials do not disappear after their use—hence, the circularity of materials is possible in principle. We can redeem materials—and exergy—at the expense of more exergy destruction. Consequently, the prime mover of circularity of materials is exergy. However, if the recovery of materials requires exergy, the pursuit for better efficiency requires materials too.

In this sense, closing the first cycle is the initial stage, and not the end goal. We know that increase the volume of recycling increases the satisfaction of society, firms, and policymakers. Yet, in most cases, such volumes are evaluated only in mass terms (i.e., tonnes/year), whereas the quality is lost in translation. In reality, reversing production processes costs more energy than production itself. Accepting and evaluating the degradation of all processes is important to understand how far removed are we from political optimism.

For example, the automobile sector generates approximately 5% of the world’s industrial waste, either from vehicles or production plants [26]. This sector is one of the largest consumers of raw materials. The demand projections on vehicle sales suggest significant increases worldwide for the next decades. By 2030, up to 1.85 billion vehicles are expected to join the current fleet [27], requiring massive amounts of raw materials. Currently, there are more than 40 different metals in a conventional passenger vehicle, but only iron, aluminum, and copper constitute more than 95% of the vehicle’s mass. Numerous other materials are necessary to build a vehicle, but they are required only in small quantities.

An assessment of current vehicles using thermodynamic rarity allows to grasp their sustainability from a raw material point-of-view [28]. We thus studied the recycling processes of metals in a common vehicle (A segment, 103 kW) to identify valuable vehicle metals and components that are not functionally recovered. We also identified which current vehicle recycling process should be improved [29].

The specific objectives of the study were to identify valuable components in terms of their raw material contents (electrical and electronics, switchers, sensors, wirings, and screens) and analyze the end-of-life (EoL) conditions of these valuable raw materials.

We found that a conventional passenger vehicle contains over 40 different metals used in more than 1000 vehicle parts (see Table 1 for the metals with their weights). The recycled metallic part constitutes 61% of the total vehicle weight. It is composed by aluminum, copper (wiring), steel, lead (battery), palladium, and platinum (in the catalytic converter). The remaining elements, whose weight does not reach 2.5% of the vehicle, are lost or not functionally recycled (when the value of a recycled product is lower than the value of the original product it was recycled from, the recycling process is called downcycling).

Minor metals in electric and electronic components and other car parts such as tantalum, silver, gold, cobalt, lithium, or tin unintentionally end up in steel-making furnaces and lose their initial functionalities. This is because there are no specific recycling processes to treat them. The mixture of different ferrous and nonferrous alloys in shredding processes is an example of downcycling because metals (with the exception of iron, aluminum, and copper) are not reused by their specific properties.

The problem of assessing metal losses and recyclability rates in mass terms, as proposed by the European Directive 2000/53/EC, is that these become irrelevant. However, these small quantities of critical metals add to a significant amount given the number of vehicles that are manufactured and sold annually. This is a serious issue not only for the automobile manufacturing sector, but also for the global economy, which relies on such materials.

If materials were assessed through thermodynamic rarity, then both the primary and minor, but valuable, metals would be considered together. On conducting an analysis as before along these lines, we found that “even if the quantity of lost or downcycled metals only represents 4.5% of the total metal weight of the vehicle, in rarity terms, this figure increases to approximately 27%.” In other words, most of the high-quality metals become functionally lost [29]. Current recycling processes of EoL vehicles are typically “aimed at isolating hazardous content, selling spare parts, recovering and recycling some regulated parts such as batteries, tires, or catalytic converters, and recycling the metallic compounds existing in the largest quantities such as steel and aluminium alloys” [30]. Recycling plants (shredders) are mainly designed to separate ferrous (steel) and non-ferrous (aluminium, copper and zinc) fractions, which are subsequently sent to smelters as secondary sources. Such an operation entails the loss of most alloy elements [31] either because they are downcycled or because they end up in the automobile shredder residue (ASR), ultimately becoming landfilled.

In future, the demand of raw and sophisticated materials for vehicles will only increase. There are, for instance, more than nine types of steel containing less than 0.5% of high-quality elements that confer special properties to the vehicle’s body and satisfy safety standards and fuel efficiency requirements [28]. Similarly, electric and autonomous vehicles will also continue to increase. In fact, plug-in hybrid vehicles and battery electric vehicles have, in rarity terms, between 1.3 and 2.3 times more impact than conventional internal combustion engines vehicles [28]. These differences can be attributed to higher use of aluminum in the latter vehicle type for light weighting—perhaps using scandium and copper for electrical and electronic components; lithium, cobalt, and nickel for lithium–ion batteries; and nickel, cobalt, lanthanum, neodymium, and praseodymium for NiMH batteries. Moreover, how will the demand for critical raw materials evolve when autonomous vehicles become smart rolling computers?

Once the car or any other product is sold, it becomes part of the societal stock and comes into use until the product arrives to its EoL, at which point it is disposed to a landfill or recycled. Its chances for a new life depend on what is most affordable: recycling or extracting more minerals from the crust. However, the price does not always reflect scarcity, and hence the conservation of scarce natural resources should not depend on markets only. An objective solution would be to compare the kW·h of rarity with the kW·h needed to obtain the same metal from a recyclate, as shown in the next section.

## 5. On Recyclability, Recoverability, and Downcyclability

When recycling, the most common industrial approach is to compare the price of the raw material against the cost of extracting it from a recyclate at the product’s EoL. In many cases, the recycling process is discarded for being uneconomic. The same comparison can be made in exergy terms with similar results. Using exergy allows us to delve deeper into the physical process. In fact, a highly mixed/alloyed object requires substantial amounts of exergy for its dismantling and metal recovery. Therefore, the industry has been in search of a “paying metal” regardless of the recoverability of additional components in the produced waste. Regularly, it becomes even more challenging to recover additional materials from such waste.

Usually, the first recycled material no longer meets the quality standards of the virgin raw material. This is the case of many metal alloys that can hardly be de-alloyed, thus reducing their further use. This common phenomenon is called downcycling. In general, the recycling processes are poorly designed. Efficiently closing the cycles requires strong industrial development in this area.

**Figure 2:**
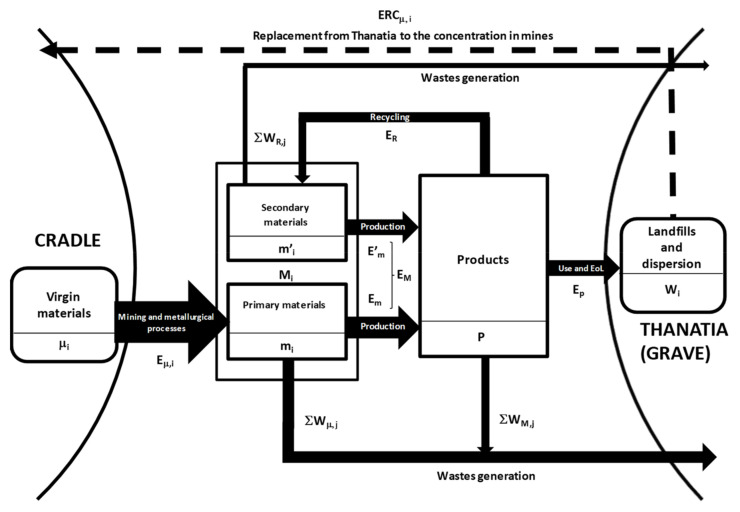
Processes and exergy costs involved in the production and end-of-life (EoL) of a product.

To make the recyclability analysis more rigorous, we elaborate the exergy indicators to be used in the assessment of the true circularity of recycling processes. Figure 2 presents a schematic diagram illustrating the indices that will be used in this section.

Accordingly, we can represent the process of manufacturing for a given product P as if it were a chemical reaction requiring an exergy cost for its manufacture, E*_M_* (kW·h)

Manufacturing: M_1_ + M_2_ + M_3_ +…+ E*_M_* → P + ∑W*_M_*_,*j*_ (1)

where M_1_, M_2_, M_3_, … are the different materials included in product P, which, in turn, will produce unavoidable waste W*_M,j_*, which is also measured in kW·h (see Figure 2). The materials of the given object can stem either from primary (m*_i_*) or from secondary raw materials (m’*_i_*)

M*_i_* = m*_i_* + m’*_i_* (2)

In turn, the energy required to produce P will differ with the primary or secondary nature of materials

E*_M_* = E*_m_* + E’*_m_* (3)

For instance, including recycled glass in the production of new glass reduces the final manufacturing energy. This is not always the case though—The final energy can increase, decrease, or remain the same if recycled materials are introduced.

Let us now analyze the recycling process, R, which implies disassembling, dismantling, dissolving, and smelting P; isolating and refining each component i; and then spending a given exergy cost E*_R_* (kW·h). This is a reverse metallurgical/industrial process that unavoidably produces waste ∑W*_R,j_*.

Full recovery: *P* + E*_R_* → m’_1_ + m’_2_ + m’_3_ +…+ ∑W*_R,j_* (4)

If the recycling process achieves the same quality as the original materials, then m’*_i_* = m*_i_*. Usually, this is not the case, and then downcycling occurs. It is important to state that in no way does reaction (4) correspond to the inverse of reaction (1). Consider, for instance, a smartphone that contains numerous assembled components manufactured from a set of intermediate materials, m_i_, which, in turn, may be composed of different chemical elements (up to 32). According to Euchems [32], these are hydrogen, lithium, potassium, calcium, magnesium, yttrium, tantalum, tungsten, cobalt, nickel, copper, gold, silver, aluminum, gallium, indium, carbon, silicon, tin, lead, arsenic, antimony, oxygen, bromine, lanthanum, praseodymium, neodymium, europium, gadolinium, terbium, and dysprosium. An example of an intermediate material is the glass used for the screen, which among others, contains silicon, oxygen, calcium, cerium, phosphorus, tin, and indium. In turn, obtaining each component material requires virgin materials, *µ_i_*, which are usually extracted from the natural environment (mineral deposits). Then, exergy, E*_µ,i_* (kW·h), is required for it to become a commodity for industrial use

Mining and casting of element *i*: *µ_i_* + E*_µ,i_* → m*_i_* + ∑W*_µ,j_* (5a)

E*_µ,i_* is the real part of the rarity of material i and is the exergy cost of extracting, beneficiating, processing, and refining raw material *i*, as explained before. This is a largescale process in current operation—that is, extract and refine virgin raw materials *μ_i_* from nature, instead of recovering them from the disassembly of EoL products. If E*_µ_* becomes the sum of E*_µi_* (kW·h), we can interpret it as the exergy required to extract from the natural environment all materials m_i_ that form object P.

*µ*_1_ + *µ*_2_ + *µ*_3_ + … + E*_µ_* → m_1_ +m_2_ + m_3_ + … + ∑W*_µ_* (5b)

The question that now arises is: Is it better to manufacture a product from virgin or from recycled materials? The recoverability condition can now be established as

- If E*_R_* < E*_µ_*, the recycling process is favorable;
- If E*_R_* > E*_µ_*, the recycling process is unfavorable.

In percentage terms, the total recoverability of product P can be defined as

RC*_M_*_,total_ = E*_µ_*/E*_R_* ∗ 100 (6)

That is, a total recoverability of 80% would mean that we are close to full recovery. In other words, with marginal technological improvements in the process of dismantling product P, it would be more favorable to reuse recycled materials than extract new raw materials. The long-term goal for the object would be to achieve an index greater than 100%, that is, the ability to recover all materials with cost efficiency to enhance the circular economy. Recoverability might also be favorable/disadvantageous depending on the amount of energy used (lesser/greater) when a recycled material is employed to manufacture a certain product P, as in the case of glass.

E*_M,S_* is the energy necessary to produce product P with primary and secondary materials compared with E*_M,V_*_,_ which is the same energy required, but from virgin materials only (E*_M,V_* = E*_m_*, as E’*_m_* = 0; Equation (3)), the condition of the use of recycled materials in a certain product would be

- If E*_R_* + E*_M,S_* < E*_µ_* + E*_M,V_*, the manufacturing process of product P with only or some recycled materials is favorable;
- If E*_R_* + E*_M,S_* > E*_µ_* + E*_M,V_*, the manufacturing process of product P with only or some recycled materials is unfavorable.

Then, the full recoverability of product P can be written as

RC*_M_*_,full_ = (E*_µ_* + E*_M,V_*)/(E*_R_*+ E*_M,S_*) ∗ 100 (7)

Importantly, the recycled material does not need to originate from the same type of material—For example, aluminum could be manufactured from aluminum can waste or from automobile scrap. Unfortunately, current technology has extremely low recoverability indices. This inefficiency can be primarily attributed to material, quality, and material efficiency losses.

First, let us consider material losses. Every process generates waste ∑W*_R,j_*. That is, the amount of material M*_i_* in product P is always greater than the quantity m’*_i_* of the dismantled product. This is the reality of the spiral economy compared with the circular economy. The cycles can never be completely closed. All processes produce products and waste. Even if the waste is reused to produce new products, new waste will be produced again. In other words,

∑W*_R,j_* > 0 (8)

Second, let us consider quality losses. The quality expressed in terms of the purity of the material may be different and usually lower with respect to the original raw material, that is, m’*_i_* ≠ m*_i_*. As explained before, this is called downcycling and we observe this in the manufacture of paper, whereby the recycling process destroys the original fiber. The resulting paper has poorer quality compared with the original natural fiber. Similarly, colored glass cannot be used to produce transparent glass. The quality of materials can be also evaluated in exergy terms, measured in kW·h/kg. Accordingly, downcycling will take place if

(9) $${m’}_{i}<m_{i}\;[\mathrm{kW}\cdot h/\mathrm{kg}]$$

If the downcycled component (such as recycled paper) can be substituted by virgin material, then the total and full recoverability conditions would still hold (Equations (6) and (7)). If not, the recoverability conditions will not be valid anymore.

Third, let us consider the loss of material efficiency because of a paying metal. Separating the component(s) of greater value in the market and discarding the remaining as non-processable waste is common industrial practice. That is, Equation (4) is replaced with Equation (10).

Recovery of paying metal: M + E*_R,k_* → m’*_k_* + ∑W*_R,j_* (10)

Because m’*_k_* has the same quality as m*_k_* (m’*_k_* = m*_k_*), the relative recovery of product P can be established.

- If E*_R,k_* < E*_µ,__k_*, the recovery process is favorable;
- If E*_R,k_* > E*_µ,__k_*, the recovery process is unfavorable.

The, we call the partial recoverability of product *P* to

RC*_M,k_* = (E*_µk_*/E*_R,k_*) ∗ 100 (11)

Thus, we gain recoverability with the paying metal at the expense of losing recyclability for the remaining components.

Under the same final conditions, if we compare the exergy needed to extract a raw material from the mine with that of the recycling process, we find that, in many cases, the recycling process becomes worthless, especially for highly mixed or alloyed products. Here, comparing the recycling exergy with its rarity than with the exergy cost of extraction and processing (embodied exergies) becomes a complementary way of approaching the need for recycling or “recyclaiming.” The greater the rarity of the given component and the more energy-saving its recovery, the greater the interest to recycle it. An example could be the gold in mobile phones. The expression would be

RC*_R_* = (1 − E*_R_* / R*_µ_*) ∗ 100 (12)

R*_µ_*= R*_µ_*_1_ + R*_µ2_* + R*_µ3_* + … = ERC*_µ_* + E*_µ_* (13)

As the rarity R*_µ_* is high for many minerals owing to their replacement cost ERC*_µ_*, the recyclability increases as the rarity of the materials used to manufacture product P. Take, for instance, the case of nickel, with an embodied exergy of 16 GJ/ton and an exergy replacement cost of 761 GJ/ton [14]. Let us now assume that recovering nickel from a given product requires 40 GJ/ton. If only E_µ_ is considered, recycling would not be favorable and Equation (12) would be negative. By way of contrast, considering the exergy replacement costs, RC_R_ would be equal to 95%, that is, the need for recyclaiming would be high. Today, very few metals are recyclaimed; among such metals, gold or precious metals are occasionally recyclaimed. The recyclaiming of other metals such as mercury, lead, arsenic, cadmium, and other heavy metals exists, but because of environmental or health issues.

In summary, using rarity instead of embodied exergy dramatically changes the result in favor of a circular economy. However, neither companies nor policymakers consider avoided costs because the present generations discard them. However, for future generations, such costs will become real costs. This predicament could be solved by an environmental tax policy that fully or partially covers avoided costs. A long-term recycling policy cannot be based on market vagaries and oscillations because raw material prices do not reflect the depletion of mineral deposits. That said, there is always an opportunity cost because avoided costs become real costs as the demand for raw materials increases. Because these costs grow exponentially with the decrease of ore grades, companies should seriously start recycling and anticipate a price increase in raw materials owing to foreseeable supply shortages.

## 6. Toward an In-Spiral Economy

Raw materials, especially critical ones, are necessary for humanity. Mobility, innovative technologies based on electronics and robotics, health, and future diets depend on such materials. The energy transition also strongly relies on raw materials. While mitigating climate change is associated with renewable energy use, the broader picture tells us that the sustainability problem is far more complex than it seems. Indeed, the energy issue is the easier part of the problem, where that of materials is more challenging. This is because the material issue is multidimensional—substitution among materials is not always possible, whereas new applications and demand increase arguably faster than the discovery of new deposits. At the same time, there are important environmental, social, and geopolitical implications of the mining industry.

Our analysis of the materials required for the energy transition shows that no future projection can neglect the associated raw materials use, especially of critical raw materials. Any projection should analyze and consider the increasing energy costs of mining and metal smelting (mostly relying on fossil fuels) because they are projected to grow in future as mines become depleted. It is imperative for all countries as well as the Earth in general to systematically account for the extraction and depletion of raw materials. That is, a global accounting that places nature in equal weight as the global economy. This global account would allow us to make decisions on policies such as new global tax structures for conserving natural resources. In fact, this was one of the objectives of the United Nations under the System of Economic and Environmental Accounts [33]. However, this scheme has had little repercussion. Even if such a move were reality, humanity cannot depend solely on extraction because material demand grows exponentially. The culture of “use and dispose” is now unsustainable, which brings into importance recycling, reusing, and reducing consumerism.

As seen in this analysis, society is far from closing even the first cycle. We are limited to a bleak future unless drastic and improbable changes are implemented: That is, the bulk of products is recycled, but not its valuable content. Current recycling processes generate more irretrievable waste as they focus on recovering the paying metal because many recycling technologies are still immature, and products are not eco-designed. Hence, it is crucial to reorient science and technological interests. Researchers are keen on producing new materials with outstanding properties, such as superalloys, nanostructures, and organometallic substances. However, very few focus on separation techniques, such as demixing, dealloying, decontaminating, or recovering basic elements to reintegrate them into the productive chain. Recovering is neither scientifically attractive nor economically profitable with respect to creating new materials and introducing them into market. Similar to how no pharmaceutical product can enter the market until its effects on health are evaluated, no new material/product should be commercialized until its expected lifecycle has been assessed. In a world with finite materials, innovations in separation warrant the same recognition as creation of new materials. In product engineering, eco-designing for recycling and disassembling is crucial, though there is also a need to extend the life of goods, search for substitutes (bio-based materials to replace rare metallic materials), and dematerialize.

We also consider it necessary to conduct “circularity audits” that disclose and quantify the quality losses in the cycle closing processes. The only way to assess the losses of quality and minimize irreversibilities is through employing thermodynamics. This science tells us that the circular economy is an elusive concept: it is as if declaring the existence of a *perpetuum mobile* fourth species. Because of the Second Law, recovering the last small particle would require an unsurmountable or even infinite exergy consumption. By recognizing that material cycles cannot be completely closed, that is, by accepting such spiral behavior, it is possible to assess how far we are from circular ideality. Just as the true efficiency of energy conversion processes is measured through exergy, the true efficiency of cycle closing processes can be measured through exergy. We can thus focus on waste and on missing factors hindering the closing of cycles, forcing us to question: What could be done with such waste? How do we minimize this waste? How do we promote eco-design to reduce waste and reuse products before destroying them? In summary, we should strive for an advanced economy focused on separating techniques and promote circularity audits based on thermodynamics.

Even if the circular economy is elusive, its central message is an urgent social need because of the current wasteful consumption culture that is depleting natural resources. The circular economy must be promoted globally. It must change the perception toward “worn out,” “old,” or “decrepit” in favor of “distinction.” Notably, exergy is a measure of distinction from commonness. Because absolute circularity does not exist, the economic practice should recognize the spiral nature of recycling processes. Therefore, distinct from a circular or linear approach, we propose an in-spiral economy. Accepting reality is not renouncing utopia—reality is much more complex and inspires novel solutions. An in-spiral economy that integrates natural resources and waste into its accounting system would stimulate the closing of multiple material cycles. It would encourage political, legal, labor, social, ecological, scientific, technological, business, behavioral, and ethical decisions in favor of greater sustainability for the current and future generations.

## Figures and Tables

**Table:** **Metals used in a vehicle that are functionally recycled:** 61% over the total vehicle weight.

Metal	Al	Cu	Fe	Pb	Pd ^1^	Pt ^2^	Total
**Weight (g)**	58,740	16,915	744,100	12,359	0.69	0.18	832,114

^1^ only the content of catalytic converters (PGMs are also used in electric and electronic components). ^2^ only the content of batteries (Pb is also used in electric and electronic components according to Annex II of Directive 2000/53/EC).

**Table:** **Metals used in a vehicle that become downcycled or lost:** 2.5% over the total vehicle weight.

**Metal**	**Ag**	**As**	**Au**	**B**	**Ba**	**Be**	**Bi**	**Cd**	**Ce**	**Co**	**Cr**	**Dy**	**Ga**	**Hg**	**In**	**La**	**Li**	**Mg**	**Mn**
**Weight (g)**	14.10	0.24	5.49	32.26	1558	0.03	9.21	0.18	5.20	8.88	4363	0.15	0.22	0.09	0.03	0.35	37.57	14496	4185
**Metal**	**Mo**	**Nb**	**Nd**	**Ni**	**Pr**	**Ru**	**Sb**	**Se**	**Sn**	**St**	**Ta**	**Te**	**Ti**	**V**	**W**	**Y**	**Zn**	**Zr**	**Total**
**Weight (g)**	232	89.0	26.62	1693	0.06	0.01	15.30	0.01	229.5	252.5	5.01	0.36	553.3	86.55	3.23	0.15	7428	7.83	35388

## References

[1] Crutzen P.J. (2002). Geology of mankind. Nature.

[2] Daly H. (2015). Economics for a Full World. Great Transit. Initiat..

[3] Valero A., Valero D.A. (2014). Thanatia: The Destiny of the Earth’s Mineral Resources.

[4] Valero A., Valero A. (2013). From Grave to Cradle. J. Ind. Ecol..

[5] Valero A., Agudelo A., Valero A. (2011). The crepuscular planet. A model for the exhausted atmosphere and hydrosphere. Energy.

[6] Valero D.A., Valero A., Gomez J.B. (2011). The crepuscular planet. A model for the exhausted continental crust. Energy.

[7] Szargut J., Morris D.R. (1985). Calculation of standard chemical exergy of some elements and their compounds based upon seawater as the datum level substance. Bull. Pol. Acad. Sci. Techical Sci..

[8] Szargut J., Ziebic A., Stanek W. (2002). Depletion of the non-renewable natural exergy resources as a measure of the ecological cost. Energ. Convers. Manag..

[9] Sciubba E. (2003). Extended exergy accounting applied to energy recovery from waste: The concept of total recycling. Energy.

[10] Dewulf J., Böch M.E., Meester B.D., der Vorst G.V., van Langenhove H., Hellweg S., Huijbregts M.A. (2007). Cumulative Exergy Extraction from the Natural Environment (CEENE): A comprehensive Life Cycle Impact Assessment method for resource accounting. Environ. Sci. Technol..

[11] Dewulf J., der Vorst G.V., Versele N., Janssens A., Langenhovea H. (2009). Van Quantification of the impact of the end-of-life scenario on the overall resource consumption for a dwelling house. Resour. Conserv. Recycl..

[12] Valero A., Valero A. (2012). Exergy of comminution and the Thanatia Earth’s model. Energy.

[13] Valero A., Valero A. (2015). Thermodynamic Rarity and the Loss of Mineral Wealth. Energies.

[14] Valero D.A., Valero A., Dominguez A. (2013). Exergy Replacement Cost of Mineral Resources. J. Environ. Account. Manag..

[15] Valero A., Valero A., Domínguez A. (2017). The thermodynamic rarity concept for the evaluation of mineral resources. Green Energy and Technology.

[16] Valero A., Valero A., Calvo G., Ortego A. (2018). Material bottlenecks in the future development of green technologies. Renew. Sustain. Energy Rev..

[17] United Nations Climate Change Secretariat (2015). Paris Agreement. http://unfccc.int/files/paris_agreement/application/pdf/qa_paris_agreement_entry_into_force.pdf.

[18] IEA (2017). Energy Technology Perspectives 2017—Catalysing Energy Technology Transformations.

[19] Valero A., Valero A., Calvo G., Ortego A., Ascaso S., Palacios J.-L. (2018). Global material requirements for the energy transition. An exergy flow analysis of decarbonisation pathways. Energy.

[20] Mudd G.M. (2007). An analysis of historic production trends in Australian base metal mining. Ore Geol. Rev..

[21] Mudd G.M. Sustainable Mining: An evaluation of changing ore grades and waste volumes. Proceedings of the International Conference on Sustainability Engineering & Science.

[22] Calvo G., Mudd G., Valero A., Valero A. (2016). Decreasing Ore Grades in Global Metallic Mining: A Theoretical Issue or a Global Reality?. Resources.

[23] Jose-Luis P., Abadias A., Valero A., Valero A., Reuter M. (2019). The energy needed to concentrate minerals from common rocks: The case of copper ore. Energy.

[24] Palacios J.-L., Fernandes I., Abadias A., Valero A., Valero A., Reuter M.A. (2019). Avoided energy cost of producing minerals: The case of iron ore. Energy Rep..

[25] Palacios J.-L., Abadias A., Valero A., Valero A., Reuter M.A. (2019). Producing metals from common rocks: The case of gold. Resour. Conserv. Recycl..

[26] Zorpas A.A., Inglezakis V.J. (2012). Automotive industry challenges in meeting EU 2015 environmental standard. Technol. Soc..

[27] Simic V., Dimitrijevic B. (2013). Risk explicit interval linear programming model for long-term planning of vehicle recycling in the EU legislative context under uncertainty. Resour. Conserv. Recycl..

[28] Ortego A., Valero A., Valero A., Restrepo E. (2018). Vehicles and Critical Raw Materials: A Sustainability Assessment Using Thermodynamic Rarity. J. Ind. Ecol..

[29] Ortego A., Valero A., Valero A., Iglesias M. (2018). Downcycling in automobile recycling process: A thermodynamic assessment. Resour. Conserv. Recycl..

[30] Andersson M., Ljunggren Söderman M., Sandén B.A. (2017). Are scarce metals in cars functionally recycled?. Waste Manag..

[31] Ohno H., Matsubae K., Nakajima K., Nakamura S., Nagasaka T. (2014). Unintentional flow of alloying elements in steel during recycling of end-of-life vehicles. J. Ind. Ecol..

[32] EUCHEMS Element Scarcity—EuChemS Periodic Table. https://www.euchems.eu/euchems-periodic-table/.

[33] United Nations (2012). System of Environmental-Economic Accounting: A Central Framework.

